# Supplementary material for: Building inclusive rehabilitation education: A scoping review protocol on EDI approaches and impact
Source: PLoS One. 2025 Apr 30;20(4):e0322110. doi: 10.1371/journal.pone.0322110 (PMC12043116; doi:10.1371/journal.pone.0322110)
Supplement: Attachment — Submitted filename: Response Letter.docx [file pone.0322110.s004.docx]

Dear Dr. De Silva,

We sincerely appreciate the time and effort that you and the reviewers have dedicated to evaluating our manuscript, "Building Inclusive Rehabilitation Education: A Scoping Review Protocol on EDI Approaches and Impact" (PONE-D-24-54307). We are grateful for the constructive feedback and have carefully revised our manuscript to address all concerns raised.

Below, we provide a point-by-point response to the reviewers’ comments and outline the corresponding revisions made to the manuscript.

**Reviewer #1 Comments:**

1. **Value of Using Policy Documents and Reports** Response: We acknowledge the reviewer's concern regarding the inclusion of policy documents and reports that may not directly examine the impact of DEI interventions in a research setting. We have now clarified in the manuscript that these sources will be used to provide contextual background, identify existing frameworks, and complement findings from empirical studies. We have also stated that these sources will be critically appraised to assess their relevance to our research objectives. Revision: Added clarification in the methodology section regarding the rationale for including policy documents and reports.
2. **Use of MMAT for Mixed Methods Studies** Response: We agree that employing a quality assessment tool for mixed-methods studies would enhance the rigor of our study. We have now specified that the Mixed Methods Appraisal Tool (MMAT) will be used for the evaluation of mixed-methods studies included in our review. Revision: Added mention of MMAT in the quality appraisal section.
3. **Community of Practice and Information Sources** Response: We appreciate the reviewer’s feedback regarding the role of the community of practice. While their primary role is to provide insights on dissemination, we also seek their expertise in identifying emerging literature or unpublished works relevant to our study. However, we acknowledge the reviewer’s concern and have revised our manuscript to ensure this aspect does not compromise replicability. Revision: Clarified the role of the community of practice in the discussion section.

**Reviewer #2 Comments:**

1. **Stakeholder Involvement and Ethics** Response: We recognize the need to clarify the distinction between stakeholder involvement and human participant research. Our engagement with rehabilitation educators, students, and patient partners is limited to seeking their perspectives on dissemination strategies and refining the research approach. This does not involve direct human subject research as defined by ethics boards. Revision: Clarified the scope of stakeholder involvement in the ethics section. We write “Stakeholder involvement in this study is limited to consultation on knowledge dissemination strategies and interpretation of findings, rather than participation in data collection or research activities. As such, this engagement does not meet the threshold for requiring ethics approval.”
2. **Inter-Rater Reliability in Reviewing Grey Literature** Response: We acknowledge the importance of inter-rater reliability for reviewing grey literature. We have now specified that two independent reviewers will assess grey literature sources, and inter-rater reliability will be measured using Cohen’s kappa. Revision: Added details on inter-rater reliability assessment in the methodology section.
3. **Time Period for Review** Response: We appreciate the reviewer’s suggestion and have now specified the time frame for literature inclusion (e.g., studies published from 2000 onwards) to capture recent developments in inclusive rehabilitation education. Revision: We write “Searches will not be limited by a time period.”
4. **Clarification on Multi-Method Approach** Response: We recognize the need for a clearer definition of our multi-method approach. We have now elaborated on how findings from the scoping review will be synthesized with stakeholder engagement insights to inform best practices and recommendations. Revision: Provided a more detailed explanation of the multi-method approach in the methodology section.
5. **Definition of Key Terms** Response: We have expanded our definitions of key terms such as "equity, diversity, and inclusion (EDI)" and "rehabilitation education" to ensure consistency and clarity throughout the manuscript. Revision: Added definitions in the introduction and methodology sections.

**Additional Revisions as per Journal Requirements:**

- Ensured consistency between the online submission form abstract and manuscript abstract.
- Reviewed the manuscript for clarity, consistency, and adherence to PLOS ONE formatting guidelines.

We appreciate the opportunity to improve our manuscript and look forward to your further feedback. Thank you for your consideration.

Sincerely,

Kristina Kokorelias, PhD
